# Analysis of risk factors contributing to neonatal pneumonia in low birth weight neonates

**DOI:** 10.3389/fped.2025.1620077

**Published:** 2025-09-24

**Authors:** Xiaoli Xu, Yongmin Deng, Jingjing Han, Jing Wang, Rui Huang, Xiaoyan Zhao

**Affiliations:** Department of Pediatrics, The First Hospital of Hebei Medical University, Shijiazhuang, Hebei, China

**Keywords:** neonatal pneumonia, low birth weight, risk factors, small-for-gestational-age, prematurity

## Abstract

**Objective:**

The current study aims to investigate the high-risk determinants associated with the occurrence of pneumonia in low birth weight (LBW) neonates.

**Methods:**

A retrospective case-control study was conducted at The First Hospital of Hebei Medical University, a tertiary care center in Shijiazhuang, China, for neonates born between January 2019 and December 2023. From a cohort of 230 LBW neonates admitted to the NICU, 90 neonates diagnosed with pneumonia were designated as the observation group, while 50 LBW neonates without pneumonia were selected as the control group. Statistical hypothesis testing was employed for data analysis, including univariate and multivariable logistic regression analyses.

**Results:**

Univariate analysis identified several significant risk factors for neonatal pneumonia, including low birth weight, prematurity (gestational age <37 weeks), small-for-gestational-age (SGA) status, neonatal anemia, patent ductus arteriosus, neonatal hyperbilirubinemia, maternal hypothyroidism during pregnancy, and prenatal infection (*P* < 0.05). Multivariable logistic regression analysis that included all significant univariate predictors revealed that birth weight (OR for <1,600 g vs. ≥2,200 *g* = 7.112, 95% CI: 1.650–30.651) and small-for-gestational-age status (OR = 2.598, 95% CI: 1.152–5.859) remained as the sole independent risk factors for neonatal pneumonia in LBW neonates.

**Conclusion:**

Birth weight and small-for-gestational-age status are independent risk factors for neonatal pneumonia in low birth weight neonates. SGA neonates born at very early gestational ages (<32 weeks) represent a particularly high-risk subgroup.

## 1 Introduction

Neonatal pneumonia, a prevalent and severe lower respiratory tract infection among neonates, arises from a multitude of contributing factors (1). In China, this disease has persistently featured prominently within the spectrum of neonatal illnesses over the last two decades. On a global scale, lower respiratory infections, including pneumonia, remain a leading cause of neonatal mortality, accounting for hundreds of thousands of deaths annually, with the vast majority occurring in developing nations (2, 3). The disease is characterized not only by its high prevalence but also by its considerable mortality rate. Reports indicate that pneumonia is a primary contributor to the nearly 2.4 million neonatal deaths worldwide each year, positioning it as a predominant cause of perinatal mortality (4). Low birth weight (LBW) neonates are a particularly vulnerable population, characterized by immature immune systems, underdeveloped organ functions, and a higher susceptibility to infections compared to their normal-weight counterparts. These inherent vulnerabilities may lead to a distinct profile of risk factors and more severe outcomes for neonatal pneumonia. Consequently, investigating the determinants of pneumonia in low birth weight neonates is crucial for the development of effective prevention and treatment strategies aimed at mitigating morbidity and mortality in this vulnerable group.

While scientific communities have explored various risk factors associated with neonatal pneumonia, there remains a paucity of research specifically addressing the risk profile of pneumonia in low birth weight neonates, particularly within our local context. This study, therefore, performs a subanalysis of a cohort of low birth weight neonates from a tertiary maternal and child health hospital. The primary objective is to identify and analyze the high-risk factors for neonatal pneumonia in this specific population. The findings are intended to inform clinical practices by aiding in the formulation of targeted prevention and control measures. Furthermore, the study seeks to contribute to the scientific foundation necessary for the optimal allocation of healthcare resources, enhancement of clinical management of neonatal diseases, and advancement of preventive healthcare strategies for women and children.

## 2 Materials and methods

### 2.1 Research subjects and study design

**Figure 1:**
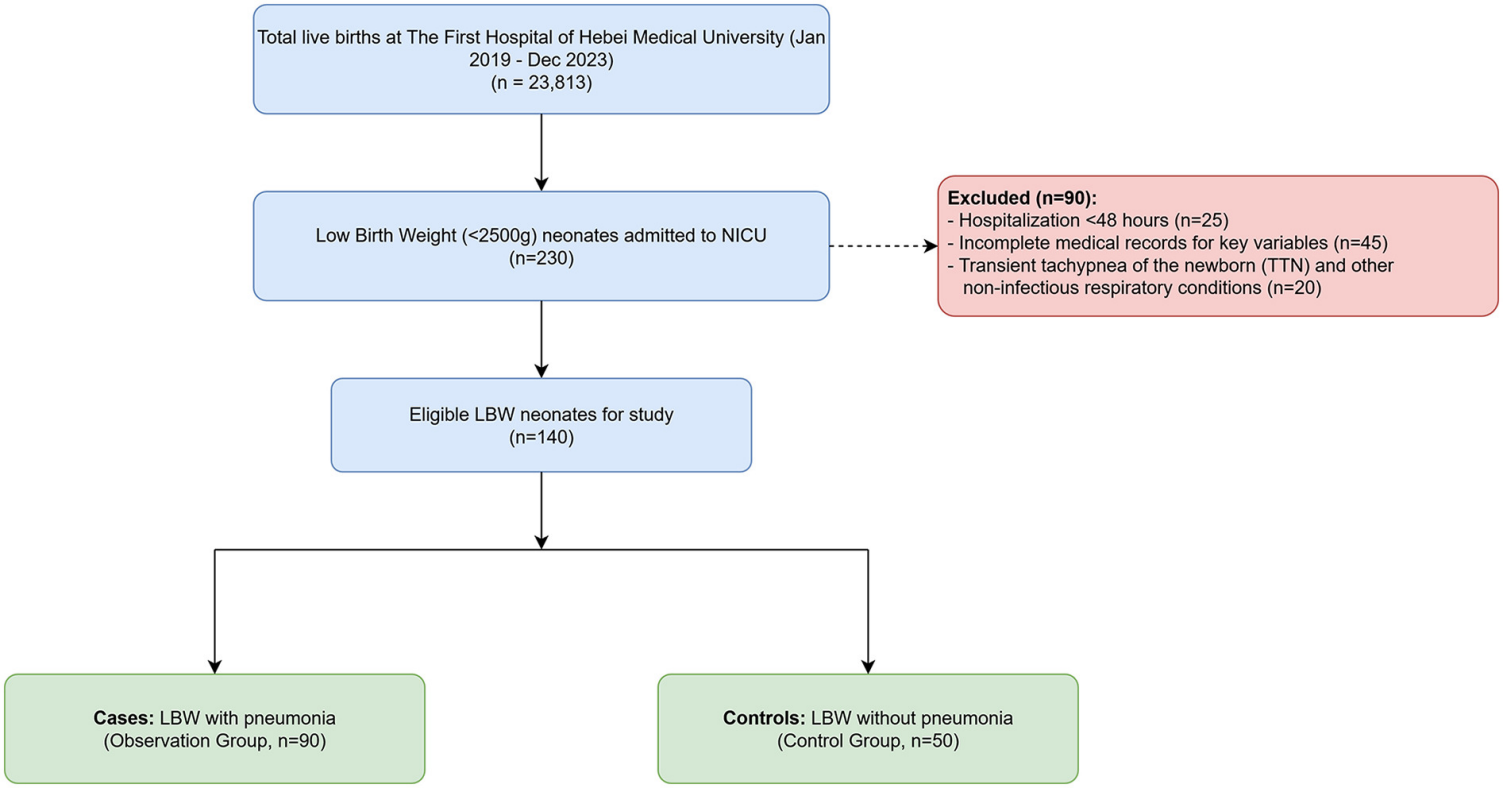
Flowchart of study participant selection.

This retrospective case-control study was conducted at the Department of Pediatrics of The First Hospital of Hebei Medical University, a tertiary maternal and child health hospital in Shijiazhuang, China. We reviewed the medical records of all neonates born between January 2019 and December 2023. Of the 23,813 live births during this period, a total of 230 low birth weight (LBW, birth weight <2,500 g) neonates were admitted to the Neonatal Intensive Care Unit (NICU). From this cohort, we selected 140 neonates who met the study's inclusion criteria. The patient selection process is detailed in Figure 1. The study population was divided into two groups: 90 LBW neonates diagnosed with neonatal pneumonia were assigned to the observation (case) group, and 50 LBW neonates without pneumonia, admitted for other reasons such as hyperbilirubinemia or feeding intolerance, were assigned to the control group. The resulting 90:50 case-to-control ratio reflects the availability of eligible subjects who met the study's inclusion and exclusion criteria during the retrospective review period.

#### 2.1.1 Study population inclusion criteria

1. Admitted to the NICU within 28 days of birth.
2. Birth weight <2,500 g., defined according to World Health Organization (WHO) criteria (5).
3. Complete clinical data available for analysis.
4. Hospitalization ≥ 48 h.

#### 2.1.2 Case and control group definitions

**Case Group (Observation Group) Definition:** LBW neonates meeting the above criteria who also conformed to a diagnosis of neonatal pneumonia, requiring the presence of characteristic clinical signs (e.g., tachypnea, grunting, retractions) combined with radiological evidence of pneumonia on a chest x-ray, as interpreted by two independent pediatric radiologists. The clinical presentations, based on established guidelines (5), were categorized as follows:

1. **Intrauterine infectious pneumonia:** Characterized by onset of respiratory symptoms at birth or within the first few hours of life, often associated with maternal risk factors like chorioamnionitis, maternal fever, or prolonged rupture of membranes.

2. **Infectious pneumonia during delivery:** Typically presenting within the first 72 h of life (early-onset neonatal pneumonia), commonly resulting from aspiration of infected maternal vaginal or rectal flora during passage through the birth canal

3. **Postnatal infectious pneumonia:** Bacterial pneumonia presents with severe toxic symptoms, including tachypnea, nasal flaring, and retractions, and is prone to systemic infections.

**Control Group Definition:** LBW neonates meeting the study population inclusion criteria who did not have neonatal pneumonia during their hospital stay. Additionally, neonates with a confirmed diagnosis of sepsis were excluded from the control group to ensure they were free from major systemic infections.

#### 2.1.3 Exclusion criteria

1. Hospitalization <48 h.
2. Primary diagnosis of transient tachypnea of the newborn (TTN) and other non-infectious respiratory conditions, such as respiratory distress syndrome without co-existing pneumonia.
3. Incomplete medical records for key variables.

### 2.2 Variables in analysis

**Neonatal-related factors:** Gender, birth weight, gestational age (weeks), postnatal age, Apgar score (5 min), delivery method, prematurity, small-for-gestational-age (SGA) status, singleton, firstborn, anemia, central atrial septal defect, patent ductus arteriosus, neonatal hyperbilirubinemia, umbilical cord abnormalities, neonatal sepsis, discharge outcome, and length of hospital stay.

**Maternal-related factors:** Maternal age, premature rupture of membranes, fetal growth restriction, intrauterine distress, chorioamnionitis, hypothyroidism during pregnancy, placental abruption, gestational diabetes, gestational hypertension, preeclampsia, anemia, and prenatal infections.

### 2.3 Quality control

Data were sourced from the electronic medical records of the hospital's Medical Records Workstation. Information on research subjects was collected and entered by two physicians to ensure data authenticity and checked for consistency by a third researcher.

### 2.4 Statistical methods

Statistical analysis was performed using SPSS 25.0. Measurement data were expressed as mean ± standard deviation $\left(\bar{x}\pm s\right)$, and categorical data were expressed as frequencies and percentages [*n* (%)]. The normality of continuous data was tested using the Kolmogorov–Smirnov Test. For normally distributed data, the two-independent samples *t*-test was used for comparison between groups. For non-normally distributed data, the non-parametric Wilcoxon rank-sum test was used. Statistical inference for categorical data was performed using the *χ*^2^ test or Fisher's exact test where appropriate.

Binary multivariable logistic regression analysis was applied to analyze risk factors for pneumonia in low birth weight neonates, with odds ratios (OR) and 95% confidence intervals (CI) used to evaluate the risk of each influencing factor. The Wald test was employed for risk difference testing. Variables with *P* < 0.10 in the univariate analysis were considered for inclusion in the multivariable model. To assess the independent effect of each potential factor, all variables found to be significant in the univariate analysis were entered into the multivariable model simultaneously using the “Enter” method. Collinearity diagnostics were performed for variables entering the model; the variance inflation factor (VIF) for gestational age was >5, indicating significant collinearity with birth weight. Consequently, gestational age was excluded from the final model to ensure stability. Birth weight was categorized based on clinically established thresholds for very low birth weight (<1,500 g, which we expanded to <1,600 g for sample size) and low birth weight, a common practice in neonatal research (6). Maternal age was included in the model as a potential confounder. The significance level was set at *α* = 0.05, and all statistical analysis results with *P* < 0.05 were considered statistically significant.

A formal *a priori* sample size calculation was not performed due to the retrospective nature of the study design. Instead, all eligible patients from the defined period were included. However, a *post-hoc* power analysis revealed that with our sample of 90 cases and 50 controls, the study achieved over 80% power to detect an odds ratio of 2.5 for the primary risk factors at a significance level of *α* = 0.05. The final regression model showed good calibration (Hosmer-Lemeshow test *P* = 0.51) and discrimination [Area Under the Curve (AUC) = 0.83, 95% CI: 0.76–0.90], with a Nagelkerke *R*^2^ of 0.36, suggesting a moderate explanatory power. Cases with significant missing data for the key variables under investigation were excluded from the analysis, as detailed in the exclusion criteria.

## 3 Results

### 3.1 Types of neonatal pneumonia

Among the 90 cases of neonatal pneumonia, 25 (27.8%) were classified as intrauterine infectious pneumonia, 41 (45.5%) as infectious pneumonia during delivery, and 24 (26.7%) as postnatal infectious pneumonia.

### 3.2 Univariate analysis of risk factors for neonatal pneumonia

#### 3.2.1 Univariate analysis of neonatal clinical characteristics and pneumonia risk

Based on electronic medical records, potential factors influencing neonatal pneumonia were analyzed. The observation group had significantly lower birth weights compared to the control group (*P* < 0.001). As detailed in Table 1, premature neonates (gestational age <37 weeks) had a 3.89-fold higher risk of developing neonatal pneumonia compared to full-term neonates (OR = 3.89, 95% CI: 1.84–8.23, *P* < 0.001). Additionally, SGA status (OR = 2.13, 95% CI: 1.01–4.49, *P* = 0.045), neonatal anemia (OR = 4.95, 95% CI: 2.01–12.21, *P* < 0.001), patent ductus arteriosus (OR = 3.26, 95% CI: 1.51–7.05, *P* = 0.002), and neonatal hyperbilirubinemia (OR = 2.96, 95% CI: 1.25–7.03, *P* = 0.012) were identified as factors that increased the risk of neonatal pneumonia. Other clinical factors, such as Apgar scores, delivery mode, singleton/firstborn status, central atrial septal defects, and umbilical cord abnormalities, showed no significant association with neonatal pneumonia (*P* > 0.05) (Table 1).

**Table 1:** Univariate analysis of factors associated with neonatal pneumonia in low birth weight neonates.

Factor	Observation group (*n* = 90)	Control group (*n* = 50)	*χ*^2^/*Z*	*P*
Gender (M/F)	42/48 (46.7%/53.3%)	26/24 (52.0%/48.0%)	0.887	0.346
Birth Weight (g)			4.762	<0.001^a^
<1,600	19 (21.1%)	2 (4.0%)		
1,600–2,200	44 (48.9%)	17 (34.0%)		
≥2,200	27 (30.0%)	31 (62.0%)		
Gestational Age (weeks)			17.982	<0.001^a^
<37	76 (84.4%)	29 (58.0%)		
≥37	14 (15.6%)	21 (42.0%)		
Apgar Score (points)			–	0.291
≥7	88 (97.8%)	50 (100%)		
<7	2 (2.2%)	0 (0.0%)		
Delivery Mode			0.581	0.446
Vaginal Delivery	38 (42.2%)	23 (46.0%)		
Cesarean Section	52 (57.8%)	27 (54.0%)		
SGA			3.971	0.046^a^
Yes	31 (34.4%)	10 (20.0%)		
No	59 (65.6%)	40 (80.0%)		
Singleton Pregnancy			1.689	0.194
Yes	74 (82.2%)	36 (72.0%)		
No	16 (17.8%)	14 (28.0%)		
Firstborn			0.129	0.719
Yes	54 (60.0%)	28 (56.0%)		
No	36 (40.0%)	22 (44.0%)		
Anemia			13.623	<0.001^a^
Yes	37 (41.1%)	6 (12.0%)		
No	53 (58.9%)	44 (88.0%)		
Central Atrial Septal Defect			0.086	0.769
Yes	33 (36.7%)	17 (34.0%)		
No	57 (63.3%)	33 (66.0%)		
Patent Ductus Arteriosus			8.635	0.003^a^
Yes	43 (47.8%)	11 (22.0%)		
No	47 (52.2%)	39 (78.0%)		
Neonatal Hyperbilirubinemia			6.257	0.012^a^
Yes	81 (90.0%)	37 (74.0%)		
No	9 (10.0%)	13 (26.0%)		
Umbilical Cord Abnormalities			0.562	0.454
Present	13 (14.4%)	5 (10.0%)		
Absent	77 (85.6%)	45 (90.0%)		

^a^ Indicates statistically significant differences in the univariate analysis, with *P* < 0.05.

#### 3.2.2 Univariate analysis of maternal clinical characteristics and pneumonia risk

Comparison of maternal clinical characteristics between the two groups revealed that maternal hypothyroidism during pregnancy (OR = 2.91, 95% CI: 1.01–8.39, *P* = 0.041) and maternal prenatal infections (OR = 5.71, 95% CI: 1.11–29.35, *P* = 0.024) were significant risk factors for neonatal pneumonia in low birth weight neonates (*P* < 0.05). Other factors, such as maternal age, premature rupture of membranes, fetal growth restriction, fetal distress, maternal chorioamnionitis, placental abruption, gestational diabetes, and maternal anemia, demonstrated no statistically significant differences between the groups (*P* > 0.05) (Table 2).

**Table 2:** Univariate analysis of maternal risk factors associated with neonatal pneumonia in low birth weight neonates.

Factor	Observation group (*n* = 90)	Control group (*n* = 50)	*χ*^2^/*Z*	*P*
Age ($\bar{x}\pm s$, years)	30.72 ± 4.31	29.68 ± 4.33	0.578	0.637
Premature Rupture of Membranes			0.045	0.831
Yes	34 (37.8%)	18 (36.0%)		
No	56 (62.2%)	32 (64.0%)		
Fetal Growth Restriction			1.231	0.267
Yes	12 (13.3%)	9 (18.0%)		
No	78 (86.7%)	41 (82.0%)		
Intrauterine Distress			1.503	0.220
Yes	10 (11.1%)	3 (6.0%)		
No	80 (88.9%)	47 (94.0%)		
Chorioamnionitis			2.356	0.125
Yes	3 (3.3%)	0 (0.0%)		
No	87 (96.7%)	50 (100%)		
Hypothyroidism During Pregnancy			4.172	0.041^a^
Yes	14 (15.6%)	3 (6.0%)		
No	76 (84.4%)	47 (94.0%)		
Placental Abruption			0.004	0.948
Yes	5 (5.6%)	3 (6.0%)		
No	85 (94.4%)	47 (94.0%)		
Gestational Diabetes Mellitus			2.051	0.152
Yes	18 (20.0%)	6 (12.0%)		
No	72 (80.0%)	44 (88.0%)		
Preeclampsia			0.204	0.652
Yes	30 (33.3%)	18 (36.0%)		
No	60 (66.7%)	32 (64.0%)		
Anemia			3.357	0.067
Yes	5 (5.6%)	6 (12.0%)		
No	85 (94.4%)	44 (88.0%)		
Prenatal Infection			5.103	0.024^a^
Yes	5 (5.6%)	0 (0.0%)		
No	85 (94.4%)	50 (100%)		

^a^ Indicates statistically significant differences in the univariate analysis, with *P* < 0.05.

### 3.3 Multivariable logistic regression analysis of risk factors for neonatal pneumonia

To identify independent predictors, all variables found to be significant in the univariate analysis (SGA, neonatal anemia, birth weight, PDA, hyperbilirubinemia, maternal hypothyroidism, prenatal infection) were entered into a binary multivariable logistic regression model. The results indicated that only birth weight and SGA status remained as independent risk factors for neonatal pneumonia in low birth weight neonates, as presented in Table 3. Specifically, neonates with birth weights of 1,600–2,200 g and <1,600 g had 2.995-fold and 7.112-fold higher risks of developing pneumonia, respectively, compared to those with birth weights ≥2,200 g. After adjusting for other factors, SGA status remained an independent risk factor, increasing the odds of pneumonia by 2.598 times (OR = 2.598, 95% CI: 1.152–5.859_, *P* = 0.021). Other variables, such as neonatal anemia and patent ductus arteriosus, were no longer statistically significant after adjusting for birth weight and SGA.

**Table 3:** Multivariable logistic regression analysis of pneumonia in low birth weight neonates.

Factor	Regression coefficient	Standard error	Wald *χ*^2^	Adjusted OR (95% CI)	*P*
Birth Weight Group	–	–	10.115	–	0.006
<1,600 g	1.962	0.721	7.412	7.112 (1.650–30.651)	0.006
1,600–2,200 g	1.097	0.388	7.989	2.995 (1.401–6.401)	0.005
≥2,200 g (Ref.)	–	–	–	1.000	–
SGA (Yes vs. No)	0.955	0.413	5.342	2.598 (1.152–5.859)	0.021
Neonatal Anemia (Yes vs. No)	0.601	0.465	1.667	1.824 (0.735–4.529)	0.197
Patent Ductus Arteriosus (Yes vs. No)	0.472	0.420	1.264	1.603 (0.707–3.635)	0.261
Hyperbilirubinemia (Yes vs. No)	0.415	0.501	0.687	1.514 (0.570–4.022)	0.407
Maternal Hypothyroidism (Yes vs. No)	0.339	0.588	0.332	1.403 (0.443–4.445)	0.564
Prenatal Infection (Yes vs. No)	0.899	0.812	1.226	2.457 (0.501–12.051)	0.268
Constant	−1.854	0.455	16.581	–	<0.001

The model was adjusted for all significant univariate predictors simultaneously. Gestational age was excluded due to high collinearity with birth weight (VIF > 5). The final model demonstrated good calibration (Hosmer-Lemeshow test *P* = 0.51) and discrimination (AUC = 0.83).

### 3.4 Comparison of gestational age distribution in SGA neonates

The distribution of gestational age in SGA neonates differed significantly between the two groups (*χ*^2^ = 9.862, *P* < 0.05). In the observation group, 61.3% (19/31) of SGA neonates had a gestational age <37 weeks, compared to 26.7% (8/30) in the control group. Compared to SGA neonates with a gestational age >37 weeks, those with a gestational age of <32 weeks had a significantly higher risk of pneumonia (OR = 12.55, *P* = 0.005). For SGA neonates with gestational ages of 32–37 weeks, the OR was 2.88 (*P* = 0.052), respectively (Table 4).

**Table 4:** Comparison of gestational age distribution in SGA neonates between two groups [*n* (%)].

Gestational age	Observation group (*n* = 31)	Control group (*n* = 10)	OR (95% CI)	*P*
<32 weeks	11 (35.5%)	1 (10.0%)	12.55 (1.89–83.31)	0.005
32–37 weeks	8 (25.8%)	7 (70.0%)	2.88 (0.99–8.39)	0.052
>37 weeks (Ref.)	12 (38.7%)	2 (20.0%)	1.00	–

## 4 Discussion

This study investigated the risk factors for neonatal pneumonia specifically within a cohort of low birth weight neonates at a single tertiary care center. Our multivariable analysis identified lower birth weight and SGA status as two key independent predictors of pneumonia in this vulnerable population. The finding that lower birth weight significantly increases pneumonia risk is consistent with a large body of literature (7, 8). Low birth weight neonates often exhibit poor growth and development, immature organ systems, impaired cellular and humoral immunity, and reduced adaptability to external environments, making them highly susceptible to neonatal pneumonia. For instance, the production of secretory IgA, crucial for mucosal defense in the respiratory tract, is deficient in LBW and especially preterm infants, compromising their first line of defense against pathogens (9, 10). Additionally, the respiratory systems of low birth weight neonates are less developed compared to those of normal neonates, with reduced effective gas exchange areas, poorer elastic tissue development, and less efficient gas exchange, all of which increase their vulnerability to respiratory infections.

Preterm neonates, who constitute a large proportion of LBW infants, have severely low levels of maternally-transferred immunoglobulins (IgG), compromised skin and mucosal barrier functions, and immature organ systems (11), making them prone to infections. Furthermore, preterm neonates have reduced sensitivity of respiratory chemoreceptors to CO2 and underdeveloped peripheral chemoreceptor functions, which hinder effective stimulation of respiratory movements during hypoxia, leading to decreased gas exchange. Consequently, the smaller the gestational age, the lower the air content in the lungs. Additionally, preterm neonates have fewer functionally active alveolar tissues, and if the mother has a history of perinatal infections such as premature rupture of membranes, pathogens may invade the respiratory tract. The inability to promptly eliminate secreted toxins may also stimulate immune cells to release inflammatory factors and pulmonary inflammatory cells to release oxygen free radicals, triggering oxidative stress and contributing to pneumonia (12).

SGA neonates, resulting from intrauterine growth restriction, are defined as neonates with birth weights below the 10th percentile for their gestational age. These neonates often experience chronic hypoxia and malnutrition *in utero* due to placental insufficiency (13). This chronic stress can impair the development of the immune system, leading to both quantitative and qualitative defects in T-lymphocytes and reduced phagocytic activity, rendering them more susceptible to infections postnatally (14). Our study's adjusted finding that SGA is an independent risk factor (OR = 2.598) aligns with this biological plausibility. Chronic fetal hypoxia can also lead to meconium aspiration pneumonia and, in severe cases, neonatal asphyxia (15). Our sub-analysis further revealed that within the SGA group, the risk of pneumonia was highest in neonates of the lowest gestational age (<32 weeks), highlighting a synergistic vulnerability of being both growth-restricted and extremely premature.

This study demonstrated that several factors were significant in univariate analysis, including neonatal anemia, patent ductus arteriosus, hyperbilirubinemia, maternal hypothyroidism, and prenatal infections. Although these factors did not retain significance in the final multivariable model after accounting for birth weight and SGA, their initial association is clinically relevant and supported by existing literature. For instance, neonatal anemia impairs oxygen-carrying capacity and tissue oxygenation, while also compromising immune function through impaired erythropoietin-mediated development and iron deficiency (16, 17). Neonatal hyperbilirubinemia can serve as an early indicator of underlying infection or sepsis (18, 19). Conditions like patent ductus arteriosus lead to pulmonary over-circulation, increasing susceptibility to respiratory infections (20). Maternal conditions are also crucial; for example, maternal hypothyroidism has been linked to delayed fetal lung maturation and altered surfactant protein expression, potentially increasing neonatal susceptibility to respiratory distress and subsequent infections (21). Similarly, maternal prenatal infections, even if subclinical, can trigger a systemic inflammatory response in the fetus, priming the neonatal immune system for dysregulated responses to postnatal challenges and increasing the risk of diseases like pneumonia (22). These factors likely contribute to the overall frailty of LBW neonates, making them more prone to pneumonia, even if birth weight and SGA are the dominant statistical predictors.

### 4.1 Strengths and limitations

This study has several strengths. It focuses on a high-risk and relatively understudied population—low birth weight neonates—and provides valuable data from a local, tertiary care setting. By performing multivariable analysis, we were able to identify independent risk factors, distinguishing them from confounding variables. Our statistical model demonstrated good predictive performance, enhancing the reliability of our findings.

However, several limitations must be acknowledged. First, the retrospective, single-center design may limit the generalizability of our findings. Second, the sample size, particularly in the control group and in subgroup analyses, was relatively small, which can lead to wide confidence intervals and limit statistical power, despite our *post-hoc* analysis showing adequate power for primary outcomes. The case-control ratio was unequal, reflecting the retrospective availability of data. Third, due to the retrospective nature of the study, we were unable to collect uniform data on potentially important confounders, such as the duration and type of respiratory support, maternal culture results, or detailed microbiological data from the neonates, which prevented us from analyzing the etiological agents of pneumonia. Finally, our method of handling missing data by exclusion could potentially introduce selection bias.

## 5 Conclusion

Taken together, the following conclusions can be drawn: (1) Birth weight and SGA status are independent risk factors for pneumonia in low birth weight neonates. Lower birth weight is associated with a progressively higher risk of pneumonia. (2) SGA neonates with a gestational age of <32 weeks are at the highest risk of developing pneumonia, underscoring the combined impact of growth restriction and prematurity.

Clinical healthcare professionals should enhance the management of pregnant women, provide early treatment for pregnancy-related complications, and strengthen health education for patients and their families. These measures aim to prevent preterm birth and intrauterine growth restriction, thereby improving neonatal birth weight and reducing the incidence of neonatal pneumonia.

## Data Availability

The original contributions presented in the study are included in the article/Supplementary Material, further inquiries can be directed to the corresponding author.
